# Analysis of the *B-RAF*^V600E^ mutation in cutaneous melanoma patients with occupational sun exposure

**DOI:** 10.3892/or.2014.2977

**Published:** 2014-01-14

**Authors:** SAVERIO CANDIDO, VENERANDO RAPISARDA, ANDREA MARCONI, GRAZIA MALAPONTE, VALENTINA BEVELACQUA, PIETRO GANGEMI, AURORA SCALISI, JAMES A. McCUBREY, ROBERTA MAESTRO, DEMETRIOS A. SPANDIDOS, CONCETTINA FENGA, MASSIMO LIBRA

**Affiliations:** 1 Laboratory of Translational Oncology and Functional Genomics, Section of General Pathology and Oncology, Department of Bio-medical Sciences, University of Catania, Catania 95124, Italy; 2 Occupational Medicine, Vittorio Emanuele - Policlinico Hospital, University of Catania, Catania 95100, Italy; 3 Section of Occupational Medicine, Department of the Environment, Security, Territory, Food and Health Sciences, University of Messina, Messina 98125, Italy; 4 Division of Pathology, Vittorio Emanuele - Policlinico Hospital, University of Catania, Catania 95100, Italy; 5 Unit of Oncologic Diseases, ASP-Catania, Catania 95100, Italy; 6 Department of Microbiology and Immunology, East Carolina University, Greenville, NC, USA; 7 Experimental Oncology 1, CRO National Cancer Institute, Aviano, Italy; 8 Department of Virology, Medical School, University of Crete, Heraklion 71003, Crete, Greece

**Keywords:** occupational sun exposure, melanoma, *B-RAF*^V600E^ mutations

## Abstract

Sun-exposure is one of the risk factors associated with the development of a cutaneous neoplasm. In melanoma, the Ras-Raf-MEK-ERK (MAPK) signaling pathway is constitutively activated through multiple mechanisms, including *B-RAF* mutation. It has been hypothesized that *B-RAF* mutations in melanocytic lesions arise from DNA damage induced by ultraviolet (UV) radiation. However, it is still discussed if *B-RAF* mutations are associated with melanoma patients exposed to the sun. Therefore, in the present study, the known *B-RAF*^V600E^ mutation was analysed in melanoma samples from 30 indoor and 38 outdoor workers. *B-RAF*^V600E^ mutation was detected in 52 and 73% of outdoor workers and indoor workers, respectively. Of note, this mutation was identified in 12 of 14 (85%) melanoma of the trunk diagnosed in indoor workers and in 9 of 19 (47%) samples from outdoor workers (p=0.03). By analyzing melanomas of other body sites, no statistical difference in the frequency of *B-RAF*^V600E^ mutation was identified between the groups of workers. It appears that the mutation detected among indoor workers may be associated with a recreational or intermittent exposure to the sun, as usually the trunk is a sun-protected body site. Overall, these data indicate that the *B-RAF*^V600E^ mutation detected in melanoma is not associated with a chronic exposure to the sun. Mutations detected in other genes may also contribute to melanoma development in the subset of patients exposed to UV radiation.

## Introduction

Cutaneous melanoma (CM) is the most aggressive form of skin cancer. Its incidence has increased dramatically worldwide over the last 50 years (1). In Europe the highest incidence rates have been reported in Scandinavia (ca. 15 cases per 100,000 inhabitants and year) and the lowest in Mediterranean countries (ca. 5–7 cases per 100,000 inhabitants and year). In a worldwide comparison, the highest incidence rates have been reported in Australia (40–60 cases per 100,000 inhabitants and year) (2,3). Epidemiologic studies suggest that melanoma is determined by a complex model of pathways that are activated by many factors including genetic factors, phenotypic characteristics, number of melanocytic nevi, anatomic sites (lower limbs in females, posterior trunk in males), family history of melanoma, and the interplay with environmental factors, in particular for cutaneous melanoma, intense and intermittent exposure to ultraviolet (UV) radiation, represent the main risk factors (4). It is well-established and recognized that a genetic predisposition exists for the development of melanoma and nevi, especially the clinically atypical variants (5). CM results from uncontrolled melanocytic proliferation, melanin-producing cells located in the basal layer of the epidermis where they have a protective role against UV radiation for the skin by distributing melanin pigment from melanosomes to keratinocytes (5). Several studies of outdoor workers have shown an excess risk of melanoma and other skin cancers (6–10). The UVB component of the solar spectrum is the main source of risk for the development of a cutaneous neoplasm. Moreover, the cumulative lifetime dose of UVB radiation seems to be the most important factor for determining carcinogenic potential (3). Intense sun exposure leads both to DNA damage and to immunosuppression, which together are held to mediate carcinogenesis, while photo-adaptation is thought to reduce DNA damage (11). Solar radiation is a highly prevalent occupational exposure in farm, fishery and construction workers, letter carriers, gardeners, lumbermen, skiing instructors and mountain guides (12). Sun-exposed workers in the EU range from 29% in Germany to 51% in Greece and 39% in Italy (3).

In melanoma, the Ras-Raf-MEK-ERK (MAPK) signaling pathway is constitutively activated through multiple mechanisms. Mutations of *B-RAF* have been proposed to contribute to melanoma development. V600E accounts for >60% of *B-RAF* mutations in melanoma and causes a substantial increase in B-Raf kinase activity (13). It has been suggested that *B-RAF* mutations in melanocytic lesions arise from DNA damage induced by UV radiation (14). However, it is still unclear if the *B-RAF*^V600E^ mutations detected in melanoma patients results from their exposure to the sun. Therefore, the *B-RAF*^V600E^ mutation was analysed in melanoma samples from patients with indoor and outdoor occupational activity.

## Patients and methods

### Patients

The subjects enrolled in this study included 68 consecutive patients diagnosed with cutaneous melanoma between October 1999 and June 2010. Tumor biopsy-specimens were isolated from 52 males and 16 females having at least 10 years of work history before the diagnosis of melanoma. All melanoma samples were collected by the Department of Bio-medical Sciences, University of Catania, Catania, Italy. The local scientific ethics committee approved all the procedures. The patients provided a written informed consent for the study. Medical files of each patient were analyzed for their occupational activity. Accordingly, patients were divided in two groups on the basis of their indoor or outdoor activity. Thirty-eight patient were outdoor workers, while 30 were indoor workers. In the group of outdoor workers there were: 11 farmers, 14 construction workers, 10 road paving workers, 2 beach attendant and 1 fisher man. In the group of indoor work there were: 11 office workers, 12 teachers, 1 Ph.D. student, 4 factory workers and 2 physicians. The duration of sun exposure for all outdoor workers was estimated as >6 h per day. Clinical patient characteristics are reported in Table I. DNA was isolated from each melanoma sample with the QIAgen Tissue kit (Qiagen, Valencia, CA, USA).

### B-RAF^V600E^ mutation analysis

All DNA samples were screened in duplicate for *B-RAF*^V600E^ mutation within exon 15 as previously described (15).

### ‘Cosmic Catalogue of Mutations in Cancer’ analysis

The website of the ‘Cosmic Catalogue of Mutations in Cancer’ (http://cancer.sanger.ac.uk/cancergenome/projects/cosmic/) was explored imputing the following key words: ‘*B-RAF*^V600E^’, ‘melanoma’, ‘skin’, ‘intermittent sun exposure’ and ‘chronic sun exposure’.

### Statistical analysis

Potential relationships between *B-RAF*^V600E^ mutation and other patient characteristics were examined by the Chi-square test or Fisher’s exact test. A p-value <0.05 by a two tailed test was defined to be statistically significant.

## Results

Clinical characteristics of melanoma patients are summarized in Table I. No differences in the main pathologic features between outdoor workers and indoor workers were observed except for the gender. The number of male outdoor workers was significantly higher compared to that of indoor workers (89 vs. 60%; p=0.004) (Table I). *B-RAF*^V600E^ mutation was detected in 42 of 68 (61%) melanoma samples. Specifically, 21 out 42 (50%) mutations were observed in melanoma of the trunk, 29% in melanoma of the head and neck site and 21% of the limbs. *B-RAF*^V600E^ mutation was detected in 20 of 38 (52%) outdoor workers and in 22 of 30 (73%) indoor workers. However, this difference was not statistically significant (Table II). In Table II, the distribution of *B-RAF*^V600E^ mutation according to the tumor sites in both groups of outdoor and indoor workers is also shown. Notably, *B-RAF*^V600E^ mutation was detected in 12 of 14 (85%) melanoma of the trunk diagnosed in indoor workers; while 47% of melanoma samples, occurred in outdoor workers, displayed this mutation in the same site (p=0.03). Similarly, higher number of mutations was observed in melanoma of the head and neck diagnosed among indoor workers when compared with those occurred among outdoor workers (78 vs. 42%). However, this difference was not statistically significant. *B-RAF*^V600E^ mutation was detected in 43% of melanoma of the limbs among indoor workers; while 86% of mutations were detected in melanoma of the same sites among outdoor workers. No statistical difference was observed (Table II).

To investigate whether *B-RAF*^V600E^ mutations may occur differentially in melanoma patients chronically exposed to sun compared with those intermittently exposed to sun, further studies were performed by analyzing the ‘Cosmic Catalogue of Mutations in Cancer’. Higher *B-RAF*^V600E^ mutation rate was detected in melanoma from patients with an intermittent exposure to sun when compared with those chronically exposed to sun (47 vs. 16%; p<0.0001) (Table III).

## Discussion

Risk factors associated with the increased incidence of melanoma are still debated. Genetic behavior and sun exposure represent the main players for cutaneous melanoma development (5,16). However, intermittent exposure to UV exposure has been demonstrated to have an increased risk of skin cancer (17).

In recent years, a growing body of evidence supports a role of MAPK pathway in melanoma cell proliferation and survival (13,18). In this disease, the most frequent genetic alteration, which accounts for >60% of melanomas is the alteration of *B-RAF*, with a glutamic acid for valine substitution at codon 600 in exon 15 (Val600Glu; *B-RAF*^V600E^); this mutation introduces a conformational change in protein structure due to glutamic acid that acts as a phosphomimetic between the Thr598 and Ser601 phosphorylation sites, leading to constitutive activation of the protein with a large increase in the basal kinase activity; the resulting hyperactivity of the MAP kinase pathway promotes tumor development (13,18).

To understand whether the B-raf^V600E^ mutation is associated to chronic or intermittent sun exposure, in the present study we analyzed this mutation in melanoma from patients with and without occupational sun exposure assuming that indoor workers have an intermittent exposure to the sun. *B-RAF*^V600E^ was mutated in 20 of 38 (52%) outdoor workers and in 22 of 30 (73%) indoor workers. Although, no statically significant difference was recorded, these data are in agreement with previous findings by Curtin *et al* (20) showing that *B-RAF*^V600E^ mutation is not associated with a chronic sun exposure. Accordingly, most recent data indicated that occupational sun exposure did not increase risk of melanoma (9,19).

Our study also analysed the *B-RAF*^V600E^ mutation according to the tumor site between indoor and outdoor workers. This mutation was detected more frequently in melanoma of the trunk from indoor workers compared with outdoor workers. We can argue that the *B-RAF*^V600E^ mutation detected among indoor workers may be associated with a recreational or intermittent exposure to the sun, as usually the trunk is a less frequently exposed body site. In fact, it was suggested that chronic exposure to the sun may induce photoadaption with increased melanisation and epidermal thickening (11). The higher frequency of *B-RAF*^V600E^ mutation melanoma of the trunk in indoor workers is in line with previous data since this mutation was detected more frequently in melanoma of the trunk (20,21). Therefore, we can speculate that the melanocytes of melanoma patients, intermittently exposed to the sun, have an increased susceptibility to proliferate and acquire *B-RAF* mutations. Further analysis were performed by exploring the Cosmic Catalogue of Mutations in Cancer confirming that higher *B-RAF*^V600E^ mutation rate is observed in melanoma from patients with an intermittent exposure to sun when compared with those chronically exposed to sun (47 vs. 16%; p<0.0001). In the study conducted by Whiteman *et al* (4) it has been shown that patients with chronic sun exposure may preferentially develop melanoma of the head and neck. Similarly, in our series melanoma of the head and neck was diagnosed in 12 outdoor workers and in 9 indoor workers, however, this difference did not reach significance due to the small number of the samples.

**Figure 1:**
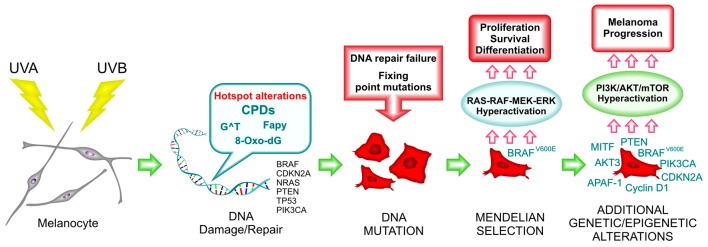
Potential mechanism of melanoma development and progression after UV-damage. UVA, Ultraviolet A; UVB, ultraviolet B; CPDs, cyclobutane pyrimidine dimers; Fapy, formamidopyrimidines; 8-oxo-dG, 8-Oxo-2′-deoxyguanosine; G^T, guanine to thymidine transversion; *B-RAF*, v-raf murine sarcoma viral oncogene homolog B; CDKN2A, cyclin-dependent kinase inhibitor 2A; NRAS, neuroblastoma RAS viral (v-ras) oncogene homolog; PTEN, phosphatase and tensin homolog; TP53, tumor protein p53; PIK3CA phosphatidylinositol-4,5-bisphosphate 3-kinase, catalytic subunit α; MITF, microphthalmia-associated transcription factor; APAF-1, apoptotic peptidase activating factor 1; AKT3, v-akt murine thymoma viral oncogene homolog 3.

Overall, these data support the notion that *B-RAF*^V600E^ mutation detected in melanoma from outdoor workers is not associated with a chronic exposure to the sun. In contrast, indoor workers, that may be exposed to intermittent sunbathing, are more susceptible to developing a melanoma harboring *B-RAF*^V600E^ mutation. These findings may have important therapeutic implications as melanoma patients with *B-RAF* mutations may benefits from *B-RAF* inhibition (18). Indication for a direct UV mutagenic effect in melanoma development remains still controversial as the nucleotide exchange detected in the *B-RAF* gene (T/A) is not classically linked to UV mutagenesis signature attributable to cytidine to thymidine (C>T) transitions. As proposed before, it is possible that B-RAF mutations could arise from error prone replication of UV-damaged DNA (14). However, a potential mechanism of melanoma development after UV-damage is described in Fig. 1. Mutations detected in other genes may also contribute to melanoma development in a subset of patients exposed to UV radiation (22).

## Figures and Tables

**Table I:** Clinical characteristics of melanoma patients.

Clinical features	Outdoor (38) n (%)	Indoor (30) n (%)	P-value
Gender			
Male	34 (89)	18 (60)	0.004
Female	4 (11)	12 (40)	
Age			
≤55	21 (55)	16 (53)	NS
>55	17 (45)	14 (47)	
Tumor type			
Primary melanoma	25 (66)	18 (60)	NS
Metastatic melanoma	13 (34)	12 (40)	
Clark’s level			
III	13 (34)	13 (43)	NS
IV	11 (29)	17 (57)	
V	1 (3)	-	
Breslow thickness			
≤2.00 mm	13 (34)	10 (33)	NS
2.01–5.00 mm	14 (37)	10 (33)	
≥5.00 mm	11 (29)	10 (33)	
Tumor site			
Trunk	19 (50)	14 (47)	NS
Head and Neck^a^	12 (32)	9 (30)	
Limbs	7 (18)	7 (23)	

a Including nose and scalp. NS, not significant.

**Table II:** Distribution of *B-RAF*^V600E^ mutation according to tumor sites in the groups of outdoor and indoor workers.

Tumor site	*B-RAF*^V600E^ mutation	Outdoor n (%)	Indoor n (%)	P-value^a^
Trunk	Yes	9 (47)	12 (85)	0.03
	No	10 (53)	2 (15)	
	Total	19	14	
Head and neck	Yes	5 (42)	7 (78)	0.18
	No	7 (58)	2 (22)	
	Total	12	9	
Limbs	Yes	6 (86)	3 (43)	0.26
	No	1 (14)	4 (57)	
	Total	7	7	
All sites	Yes	20 (52)	22 (73)	0.08
	No	18 (48)	8 (27)	
	Total	38	30	

a Fisher’s exact test, Two-tailed.

**Table III:** Distribution of *B-RAF*^V600E^ mutation according to chronic exposure to sun and intermittent exposure to sun.^a^

*B-RAF*^V600E^ mutation	Chronic exposure to sun n (%)	Intermittent exposure to sun n (%)	P-value^b^
Yes	9 (16)	30 (47)	<0.0001
No	46 (84)	34 (53)	
Total	55	64	

a Cosmic Catalogue of Mutations in Cancer analysis;

b Fisher’s exact test, Two-tailed.
